# Phosphoproteomic profiling of the hippocampus of offspring rats exposed to prenatal stress

**DOI:** 10.1002/brb3.2233

**Published:** 2021-09-14

**Authors:** Qinghong Li, Dongge Cai, Huimei Huang, Huiping Zhang, Ruimiao Bai, Xiaolin Zhao, Hongli Sun, Pei Qin

**Affiliations:** ^1^ Department of Neonatology Northwest Women's and Children's Hospital Xi'an Shaanxi P.R. China; ^2^ Department of Obstetrics and Gynecology The Second Affiliated Hospital of Xi'an Jiaotong University Xi'an Shaanxi P.R. China; ^3^ Department of Nephrology Xi'an Children's Hospital (The Affiliated Children's Hospital of Xi'an Jiaotong University) Xi'an Shaanxi P.R. China; ^4^ Shaanxi Institute for Pediatric Diseases, Xi'an Key Laboratory of Children's Health and Diseases Xi'an Children's Hospital (The Affiliated Children's Hospital of Xi'an Jiaotong University) Xi'an Shaanxi P.R. China; ^5^ Department of Anaesthesiology Xi'an Children's Hospital (The Affiliated Children's Hospital of Xi'an Jiaotong University) Xi'an Shaanxi P.R. China

**Keywords:** phosphoproteomics, prenatal stress, offspring, hippocampus, depressive‐like behaviors

## Abstract

**Introduction:**

Prenatal stress (PS) can cause depression in offspring. However, the underlying biological mechanism of these influences is still unclear. This work was implemented to investigate the molecular mechanisms of depressive‐like behavior of offspring rats insulted with PS.

**Methods:**

Relative quantitative phosphoproteomics of the hippocampus of PS susceptibility (PS‐S) and control (CON) rat offspring was performed using  liquid chromatography‐tandem mass spectrometry to confirm known pathways and to identify new mechanisms involved in depression.

**Results:**

A total of 6790 phosphopeptides, 9817 phosphorylation sites, and 2978 phosphoproteins were detected. Among the 2978 phosphoproteins, 1760 (59.09%) had more than two phosphorylated sites, the ENSRNOP00000023460 protein had more than 117 phosphorylated sites, and the average distribution of modification sites per 100 amino acids was 2.97. There were 197 different phosphopeptides, including 140 increased phosphopeptides and 57 decreased phosphopeptides in the PS‐S offspring rats, compared to the CON offspring rats. These differential phosphopeptides corresponded to 100 upregulated and 44 downregulated phosphoproteins, respectively. Gene ontology enrichment analysis revealed that these different phosphoproteins in the top five enriched terms in the cellular component, molecular function, and biological proces categories were involved in a total of 35 different phosphoproteins, and these phosphoproteins were mainly related to myelin‐, microtubule‐ and synapse‐associated proteins. The enrichment of Kyoto Encyclopedia of Genes and Genome pathways was found to be involved in many essential biological pathways, and the top five pathways included amphetamine addiction, insulin secretion, Cushing syndrome, and the circadian entrainment signaling pathway. These first five pathways were related to nine phosphoproteins, including Adcy9, Apc, Cacna1c, Camk2a, Camk2b, Camk2g, Ctnnd2, Grin2a, and Stx1a. The full data are available via ProteomeXchange with identifier PXD019117.

**Conclusion:**

We preliminarily identified 144 different phosphoproteins involved in myelin, microtubule, and synapse formation and plasticity in the hippocampus of susceptible offspring rats exposed to PS.

## 1 INTRODUCTION

Prenatal stress (PS) is defined as maternal psychological stress, depression, and anxiety during pregnancy (De Weerth, 2018). Many clinical and laboratory investigations have indicated that PS could increase the rate of depression disorder or depressive‐like behaviors of offspring (Fatima et al., 2019; Robinson et al., 2019). For example, one report indicated that maternal depression during pregnancy could increase the risk of depressive disorder by 3.4 times in adult offspring and 2.4 times in those who experienced child maltreatment, compared with nonexposed offspring (Plant et al., 2015). Data from another researcher also confirmed this result. The report indicated that antenatal depression was an independent risk factor, and offspring were 1.28 times more likely to have depression at age 18 for each standard deviation (SD) increase in maternal depression score antenatally, independent of later maternal depression(Pearson et al., 2013). The results of animal models also showed that PS could increase immobility time in the forced swim test (FST), decrease sucrose consumption in the sucrose preference test (SPT), and the total distance traveled in the open field test (OFT) of offspring (Weinstock, 2017; Zhang et al., 2019). However, the underlying biological mechanism of these influences is still unclear.

Extensive research has shown that the hippocampus is not only crucially involved in the pathological mechanism of depression but is also highly sensitive to stress (Hei et al., 2019; Krzystyniak et al., 2019; Sheline et al., 2019). PS enhanced susceptibility to depression by changing the structural and functional hippocampus of offspring (van den Bergh et al., 2018). A significant decrease in the volume and the total number of cells in the hippocampus was found in rats (Soares‐Cunha et al., 2018), and the number of neuronal processes and synaptic density in sheep (Hermes et al., 2020) exposed to PS was reduced. PS could change neurogenesis and neurodevelopmental and synaptic plasticity of the hippocampus through the hypothalamic–pituitary–adrenal axis; gamma‐aminobutyric acid; glutamate, dopamine, and serotonergic systems (Gemmel et al., 2016; Nejatbakhsh et al., 2018; Sun et al., 2013; van Bodegom et al., 2017; Zhang et al., 2017).

In recent years, some bioinformatics analyses, including metabolic and proteomic analyses, have provided a change in the biological profile of the system in the hippocampus insulted by stress. In organisms, proteins are the most important executors of physiological functions. As the most common of many posttranslational modification types, phosphorylation changes of proteins play an important role in the regulation of almost all life activities since they play a vital role in intracellular signal transduction and are involved in regulating cell cycle progression, development, response, differentiation, transformation, and metabolism.

Phosphoproteomics, as an ideal tool for the study of phosphorylation changes in proteins, has already been used to explore the mechanisms of stress‐induced depression. A recent work showed that stress changed 3988 phosphorylation sites from the medial prefrontal cortex and 3196 phosphorylation sites from the nucleus accumbens when compared to its matched control (CON), and these changes mainly focused on synaptic transmission‐related signaling and could be reversed by ketamine treatment (Xiao et al., 2020). However, changes in phosphorylation profiles in the hippocampus of offspring rats exposed to PS have not been reported at present.

To investigate the molecular mechanisms of depressive‐like behavior of offspring rats insulted with PS, we analyzed alterations in phosphoprotein profiles of offspring rats susceptible to PS and CON offspring rats using liquid chromatography‐tandem mass spectrometry (LC‐MS/MS) with TMT labeling. This study provides valuable data and novel insights for further investigations into molecular events involved in depressive‐like behavior of offspring rats subjected to PS.

## 2 MATERIALS AND METHODS

### 2.1 Animal subjects

**FIGURE 1:**
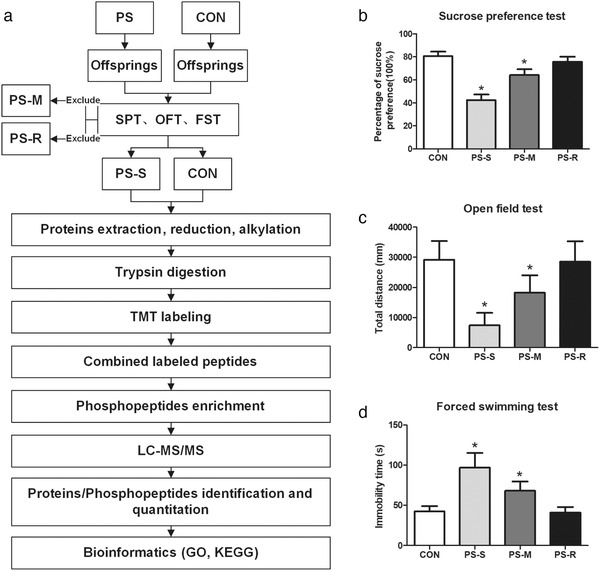
(a) Schematic workflow of the experimental design and behavioral assessment of the prenatal stress (PS) rat model. (b) Percentage of sucrose preference in the sucrose preference test. (c) Total distance in the open field test. (d) Immobility time in the forced swimming test. Values represent means ± standard deviation, *n* = 8 per group. *p < .05 versus control (CON)

In the present work, a total of 60 Sprague Dawley rats including 28 adult rats and 32 offspring male rats were used in the entire experiment. The flow chart of the experiment is shown in Figure 1. During the test, rats were maintained with a 12‐h dark/light cycle at 22°C and allowed to drink and eat *ad libitum*. Adult female (*n* = 21) and male rats (*n* = 7) were mated by 3:1 at 8:00–10:00 p.m. The next morning, positive sperm in the vagina of female rats were detected as gestational day 0. Pregnant dams were raised in a single cage and randomly divided into the PS group and the CON group. All experimental procedures were approved by the Experimental Animal Care and Use Committee of Xi'an Jiaotong University.

### 2.2 PS procedure

The prenatal restraint stress procedure was applied according to the method described in a previous article (Koehl et al., 2015). Briefly, PS dams were placed into a plastic bottle with an adjustable cap to keep the dam's head still (three times/day, 45 min/time) in a new cage. After each treatment, pregnant rats were returned to their original housing environment. In parallel, CON pregnant rats were placed in a new cage with the same design but without PS treatment. After the dams gave birth, litters were selected with the size of 6–12 pups, during the postnatal day 30–35, no more than two male pups from each litter were randomly chosen for subsequent experiment. All offspring rats underwent behavioral tests to distinguish PS susceptibility (PS‐S), PS midterm (PS‐M) and PS resistance (PS‐R), and the CON group was collected from CON dams. A total of 32 (*n* = 8 in each group) rat offspring were used in the present work.

### 2.3 SPT

SPT was applied using the method described in a previous article (Kim et al., 2012). Briefly, all offspring rats were subjected to SPT at 1 month of age. Before the test, rats were habituated to a bottle with 1% sucrose for 24 h. After deprivation of food and water for 24 h, a bottle with normal tap water and a bottle with 1% sucrose solution were provided to the rats. After 1 h, the amount of normal tap water and sucrose solution consumed was recorded, and the preference for sucrose was calculated: the preference of sucrose preference = (sucrose consumption/(sucrose consumption + normal tap water consumption) x 100%). Rats with a reduction in sucrose preference of more than 30%, compared to the average of the CON group were assigned to the PS‐S group, less than 10% were assigned to the PS‐R group, and between 10% and 30% were assigned to the PS‐M group.

### 2.4 OFT

After SPT, rats were subjected to OFT (Taimeng Software Co. Ltd., item no: OFT‐100). Briefly, rats were placed in the central grid of an open‐field apparatus, which was 74 cm wide, 62 cm long, and 51 cm high. The bottom of the box was divided into 25 equal squares, and the central nine grid was where the rats were placed. The total distance traveled was recorded by a video camera for 5 min. Feces and urine on the bottom were cleaned away with alcohol to avoid residual smells after each test.

### 2.5 FST

After the OFT, rats were subjected to the FST (Youcheng Jiaye Biotechnology Co., item no: LTDLE803/804). Briefly, the rats were gently placed into a vertical glass cylinder with water. The height of the cylinder was 50 cm, and the diameter was 20 cm. The surface of the water was 30 cm from the bottom at 30°C. Rats were habituated to the cylinder for 15 min. After 24 h, rats were placed into the cylinder again, and the immobility time within 5 min was recorded by video analysis. The immobility time was defined as no struggling or only minimal movements during swimming.

### 2.6 Tissue collection and phosphoproteomic sample preparation

After all behavioral tests, the rats were anesthetized with sodium pentobarbital (60 mg/kg, i.p.). The total brain was isolated and placed on ice, and the bilateral hippocampus was removed from the brain immediately. Then, the bilateral hippocampal tissue was cut into pieces and ground evenly and 100 mg samples were added to SDT buffer and transferred to 2 ml tubes with quartz sand (another 1/4 inch ceramic bead MP 6540‐424 for tissue samples). The lysate was homogenized by an MP homogenizer (24 × 2, 6.0 M/S, 60 s, twice). The homogenate was sonicated and then boiled for 15 min. After centrifugation at 14,000 g for 40 min, the supernatant was filtered with a 0.22 μm filter. The filtrate was quantified with the BCA Protein Assay Kit (Bio‐Rad).

Trypsin hydrolysis of the protein in each sample was performed by the FASP method (Wiśniewski et al., 2009). Protein of 200 mg for each sample was mixed with 30 μl SDT buffer (4% SDS, 100 mM DTT, 150 mM Tris‐HCl pH 8.0). The detergent, DTT, and other low‐molecular‐weight components were removed using UA buffer (8 M urea, 150 mM Tris‐HCl pH 8.0) by repeated ultrafiltration (Microcon units, 10 kD). Then, 100 μl iodoacetamide (100 mM IAA in UA buffer) was added to block reduced cysteine residues, and the samples were incubated for 30 min in darkness. The filters were washed with 100 μl UA buffer three times and then with 100 μl dissolution buffer (DS buffer) twice. Finally, the protein suspensions were digested with 4 μg trypsin (Promega) in a 40 μl DS buffer overnight at 37°C, and the resulting peptides were collected as a filtrate. The peptides of each sample were desalted on C18 cartridges (Empore™ SPE Cartridges C18 (standard density), bed I.D. 7 mm, volume 3 ml, Sigma), concentrated by vacuum centrifugation and reconstituted in 40 μl of 0.1% (v/v) formic acid. The peptide content was estimated by Ultraviolet light spectral density at 280 nm using an extinction coefficient of 1.1 of 0.1% (g/l) solution that was calculated on the basis of the frequency of tryptophan and tyrosine in vertebrate proteins. Three biological replicates for each group were performed.

Peptides (100 μg) in each sample were collected and labeled using TMT reagent according to the manufacturer's instructions (Thermo Fisher Scientific). The labeled peptides were mixed, concentrated by a vacuum concentrator, and resuspended in 500 μl 1 × DHB buffer. Then, TiO_2_ beads were added and agitated for 2 h. Centrifugation was carried out for 1 min at 5000 g, resulting in the removal of the beads. Then, 50 μl of washing buffer I was added three times, and 50 μl of washing buffer II was added three times to remove the remaining nonadsorbed material. Finally, the phosphopeptides were eluted with 50 μl of elution buffer three times, followed by lyophilization and MS analysis.

### 2.7 LC‐MS/MS

Nano‐LC‐MS/MS analysis was used to separate the peptides. Buffer A was 0.1% formic acid, and buffer B was a mixture of 84% acetonitrile and 0.1% formic acid. Ninety‐five percent buffer A was used to balance the chromatographic column. Samples were loaded onto a reversed‐phase trap column (Thermo Scientific Acclaim PepMap100, 100 μm × 2 cm, nanoViper C18, Thermo Fisher Scientific), subjected to an analytical column (Thermo Scientific Easy Column, 10 cm, ID75 μm, 3 μm, Thermo Fisher Scientific), and the flow rate was 300 nl/min controlled by IntelliFlow technology.

LC‐MS/MS analysis was performed on a Q Exactive mass spectrometer (Thermo Scientific) that was coupled to Easy nLC (Proxeon Biosystems, now Thermo Fisher Scientific) for 120 min. The mass spectrometer was operated in positive ion mode. MS data were acquired using a data‐dependent top10 method dynamically choosing the most abundant precursor ions from the survey scan (300−1800 m/z) for HCD fragmentation. The automatic gain control target was set to 3e6, and the maximum injection time was set to 10 ms. The dynamic exclusion duration was 40.0 s. Survey scans were acquired at a resolution of 70,000 at 200 m/z, the resolution for the HCD spectra was set to 17,500 at 200 m/z, and the isolation width was 2 m/z. The normalized collision energy was 30 eV, and the underfill ratio, which specifies the minimum percentage of the target value likely to be reached at the maximum fill time, was defined as 0.1%. The instrument was run with peptide recognition mode enabled.

### 2.8 Motif analysis

The modification sites and ± 6 amino acids of the modified sites were extracted to predict the possible conserved motifs using MEME software (http://meme‐suite.org/index.htm). The parameters of the software were set as follows: width was 13, the occurrence was 50, and background was Ensembl_Rattus_29107_20190628.

### 2.9 Data analysis

For all behavioral tests, data are presented as the means ± SD. Kolmogorov–Smirnov was used for normality tests. One‐way ANOVA was used for comparisons between groups. Dunnett's test was used for multiple comparisons as a post hoc analysis. *p* < .05 was set as the significance level. Statistical analysis was accomplished by Prism version 5.0 software (GraphPad Software Inc.).

For LC‐MS/MS analysis, the raw MS spectra were searched using the MASCOT engine (Matrix Science, version 2.2) embedded into Proteome Discoverer 1.4. The observed MS/MS spectra were obtained using Ensembl_Rattus_29107_20190628. An initial mass tolerance of precursor ions was set at 6 and 20 ppm, and two missed cleavages were allowed. The fixed modification was carbamidomethylation for cysteines, TMT 6plex (N‐term), TMT 6plex (K). Variable modifications were phosphorylation modifications for lysines, tyrosine, and serine. The false discovery rate of peptide spectrum matching, protein assembly, and modification identification was set at 1%. Only phosphopeptides were considered for expression analysis in at least two of the three biological replicates in at least one sample. The ratios of phosphopeptides were normalized by the median protein ratio. For TMT quantification, a fold change of ≥1.2 or ≤ 0.83 and *p* values of < .05 were defined as significantly different. The mass spectrometry proteomics data have been deposited in the ProteomeXchange Consortium via the PRIDE (Perez‐Riverol et al., 2019) partner repository with the dataset identifier PXD019117.

### 2.10 **Gene ontology (GO) and** Kyoto Encyclopedia of Genes and Genome **(KEGG) annotation**

The differentially expressed phosphoproteome was annotated using Blast2GO (Mulder & Apweiler, 2007) to be classified into different GOs. Then, KAAS software (KEGG Automatic Annotation Server: http://www.genome.jp/kegg/kaas/) was applied to map the significant differentially expressed phosphorylated proteins to the terms in the Kyoto Encyclopedia of Genes and Genomes (KEGG) database. KEGG pathway analysis was employed, and the pathways or GO terms with a *p* < .05 were considered significantly different.

To further explore the impact of differentially expressed proteins on cell physiological processes and to discover internal relations between differentially expressed proteins, enrichment analysis was performed. GO enrichment on three ontologies (biological process (BP), molecular function (MF), and cellular component (CC)) and KEGG pathway enrichment analyses were applied based on Fisher's exact test, considering the whole quantified protein annotations as a background dataset. Benjamini–Hochberg correction for multiple testing was further applied to adjust the derived *p*‐values. Only functional categories and pathways with *p*‐values under a threshold of .05 were considered significant.

### 2.11 **Hierarchical** c**lustering**

The studied relative protein expression data were used to perform hierarchical clustering analysis. For this purpose, Cluster3.0 (http://bonsai.hgc.jp/~mdehoon/software/cluster/software.htm) and Java Treeview software (http://jtreeview.sourceforge.net) were used. The Euclidean distance algorithm for similarity measurement and the average linkage clustering algorithm (clustering uses the centroids of the observations) for clustering were selected when performing hierarchical clustering. Heatmaps are often presented as a visual aid in addition to dendrograms.

## 3 RESULTS

### 3.1 Behavioral assessment of offspring rats exposed to PS

The flow chart of the present experiment is shown in Figure 1(a). SPT was used to screen offspring rats that showed a susceptibility response to PS. All results of a behavioral experiment conform to the normal distribution (Table S1–3). Figure 1(b) shows a significant difference found between groups, which was tested by one‐way ANOVA (*F* = 111.358_(3,31)_, *p* < .05). Post hoc analysis indicated the percentage of sucrose consumed in the PS‐S and PS‐M groups was significantly less than that in the CON group (all *p* < .05). Rats in the PS‐R group showed no significant difference in the percentage of sucrose consumed as compared to rats in the CON group (*p* > .05). OFT and FST were used to confirm the results of the SPT. As shown in Figure 1(c), a significant difference was found between groups, which was tested by one‐way ANOVA (*F* = 24.729_(3,31)_, *p* < .05). Post hoc analysis indicated the total distance traveled in the OFT of rats in the PS‐S and PS‐M groups was significantly less than that traveled in the CON group (all *p* < .05). Rats in the PS‐R group showed no significant difference in the total distance traveled, compared to rats in the CON group (*p* > .05). For the FST (Figure 1(d)), a significant difference was found between groups, which was tested by one‐way ANOVA (*F* = 38.540_(3,31)_, *p* < .05). Post hoc analysis indicated that the immobility time of rats in the PS‐S and PS‐M groups was significantly greater than that in the CON group (all *p* < .05). Rats in the PS‐R group showed no significant difference in immobility time, compared to rats in the CON group (*p* > .05). These results demonstrated the availability of PS models and the screening conditions of the SPT.

### 3.2 Quantitative phosphoproteomics in the hippocampus of rat offspring

**FIGURE 2:**
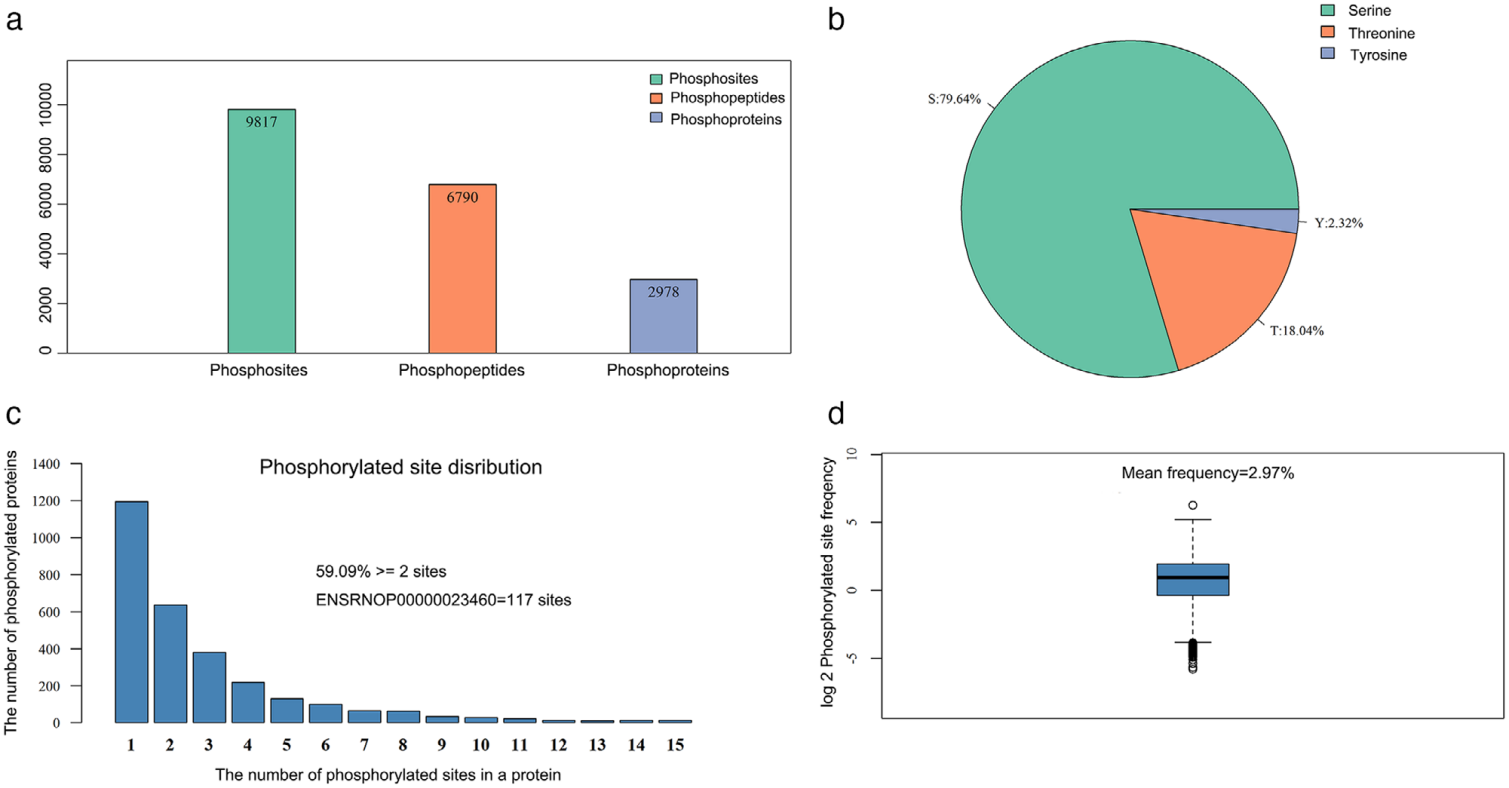
Description of phosphoproteome data. (a) Number of phosphorylation sites, phosphopeptides, and phosphoproteins identified; (b) number of phosphorylation modifications for serine, threonine, and tyrosine; (c) distribution of phosphorylated sites; and (d) frequency of phosphorylated sites

A total of 6790 phosphopeptides, 9817 phosphorylation sites, and 2978 phosphoproteins were detected in the present work (Figure 2(a)). Among the 9817 identified phosphorylation sites, 7818 (79.64%) were from phosphoserine, 1771 (18.04%) were from phosphothreonine, and 228 (2.32%) were from phosphotyrosine (Figure 2(b)). Among the 2978 phosphoproteins, 1760 (59.09%) had more than two phosphorylated sites, the ENSRNOP00000023460 protein had more than 117 phosphorylated sites (Figure 2(c)), and the average distribution of modification sites per 100 amino acids was 2.97 (Figure 2(d)).

### 3.3 Motif analysis of identification of phosphopeptides

**FIGURE 3:**
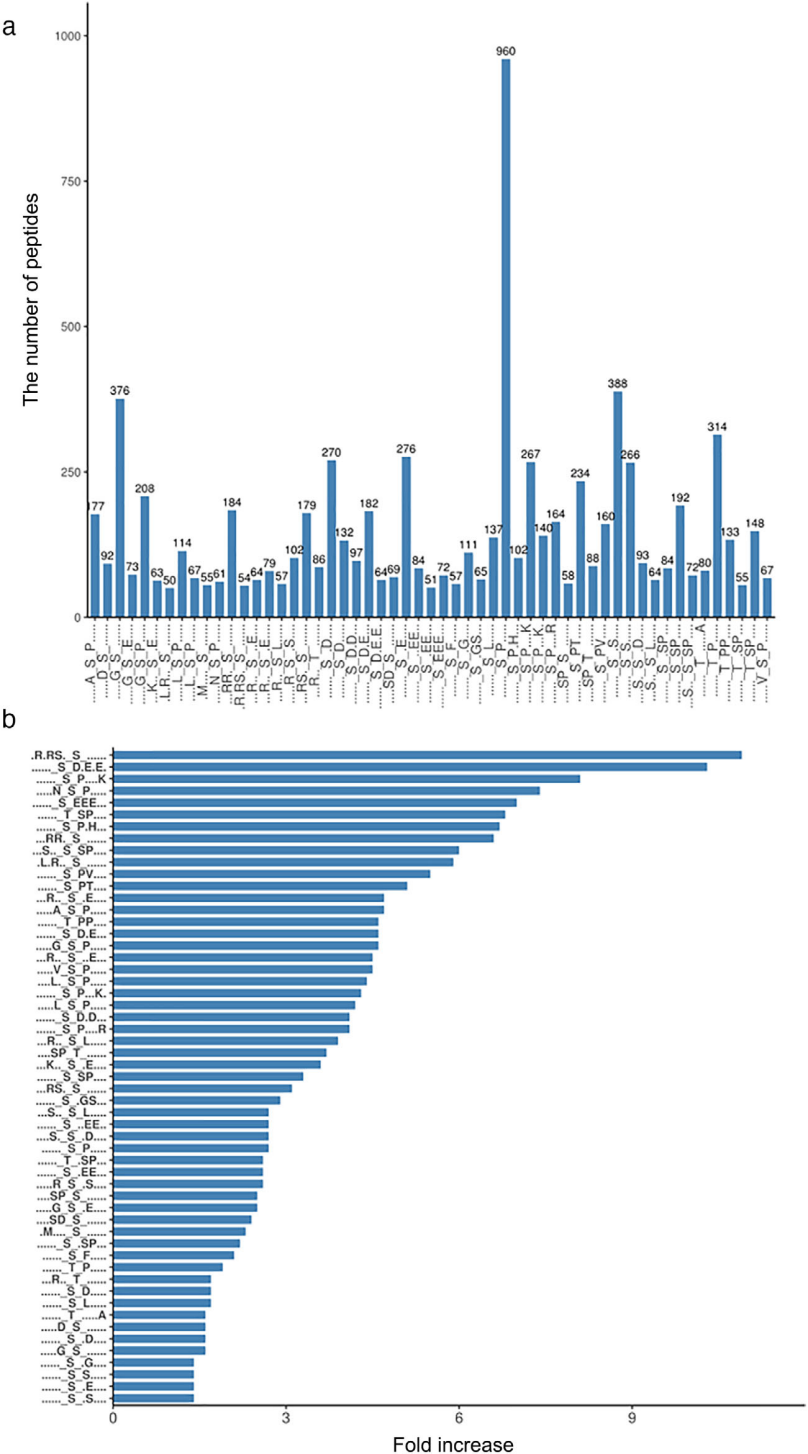
Analysis of phosphorylation sites. (a) Number of identified phosphopeptides containing phosphorylation sites in each motif, (b) enrichment of individual motifs in the differentially expressed phosphopeptides

Motif analysis was used to predict motifs by MEME software (Schwartz, 2011) in this study. In total, 48 Ser motifs and 7 Thr motifs (Table S4) were significantly enriched. These motifs exhibited different abundances; in 48 pSer motifs, motifs [……SP….] occupied the highest proportion of 960. The lowest proportion of these motifs of phosphorylated peptides was [.L.R. S……], which only accounted for 50 of all identified peptides (Figure 3(a)). In the seven pThr motifs, motifs ……TP…. occupied the highest proportion of 314, and motifs [……T. SP…] occupied the lowest proportion of 55 identified phosphorylated peptides (Figure 3(a)). We further investigated the enrichment of each motif in PS‐S rats, compared to CON rats by calculating the fold changes of the occurrence of the motifs. The results showed that the top three enriched phosphor‐serine motifs were [.R.RS. S……], [……SD.E.E.] and [….NSP….] comparison to Ensembl_Rattus_29107_20190628 (Figure 3(b)).

### 3.4 **Differential phosphopeptides between PS‐susceptible and** CON **offspring rats**

**FIGURE 4:**
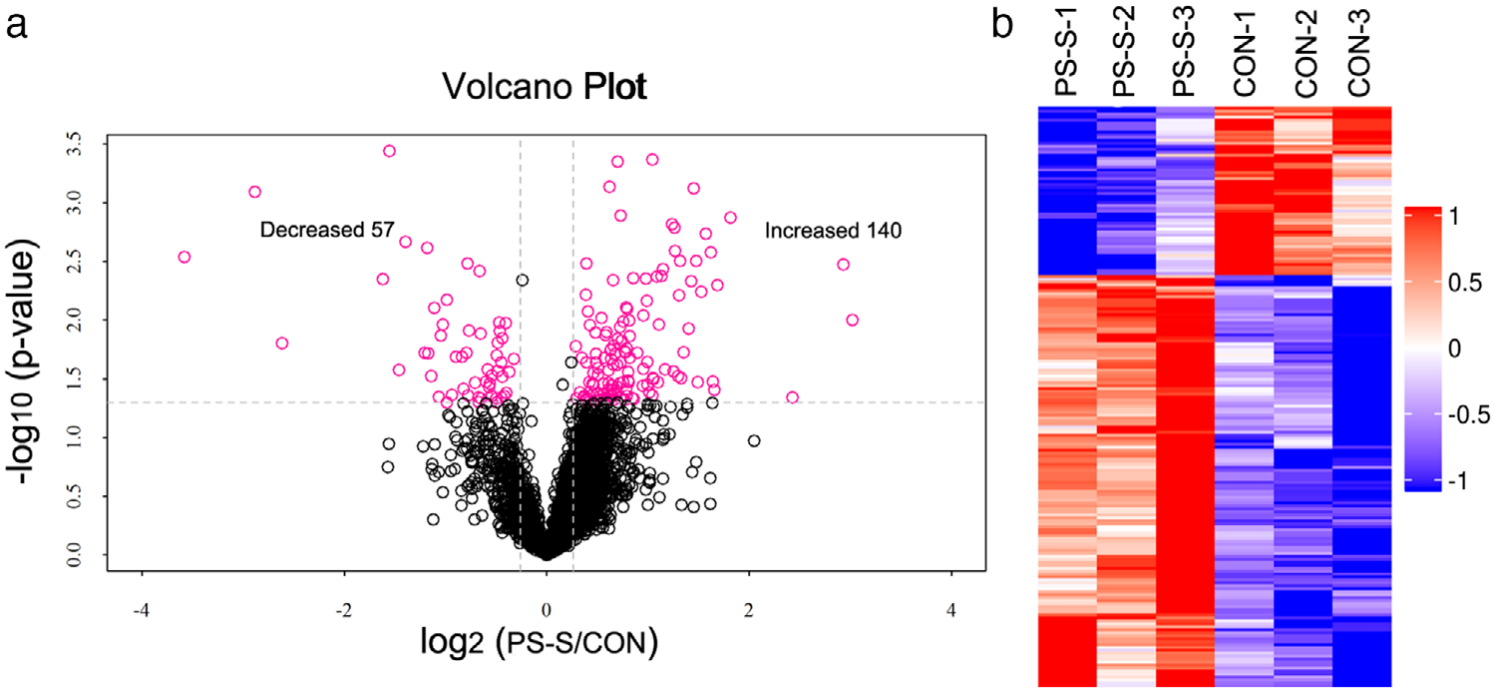
Analysis of the differentially expressed phosphopeptides from phosphoproteomic profiling. (a) Volcano plot showing variations in phosphopeptides from the hippocampus in PS susceptibility and CON offspring rats exposed to PS. The fold change log (base 2) is on the x‐axis, and the negative false log discovery rate (*p*‐value; base 10) is on the y‐axis. Significant changes in phosphopeptides are indicated in pink, and no significant changes are indicated in black. (b) Heatmap clustering of the differentially expressed phosphopeptides in the two groups. X‐xis: the hierarchical clusters for samples. Y‐axis: the hierarchical clusters for phosphopeptides. Hierarchical clustering analysis was performed on the log 2 expression of differentially expressed modified phosphopeptides displaying similarity among treatments. Red represents significantly increased modified phosphopeptides. Blue represents significantly decreased modified phosphopeptides. Gray represents the modified phosphopeptides having no quantitative information

After setting the cutoff fold change to > 1.2 or < 0.83 and *p* < .05, which are the criteria commonly used in TMT labeling data analysis, we identified 140 phosphopeptides were increased and 57 phosphopeptides were decreased in PS‐S offspring rats, compared to CON offspring rats. These differential phosphopeptides corresponded to 100 upregulated and 44 downregulated phosphoproteins in PS‐S offspring rats, respectively. The magnitude and significance of changes in the protein phosphorylation level between PS‐S and CON offspring rats are presented in a volcano plot (Figure 4(a)) and hierarchical clustering heat maps (Figure 4(b)).

### 3.5 GO and KEGG analysis of differentially expressed phosphopeptides corresponding to phosphoproteins

**FIGURE 5:**
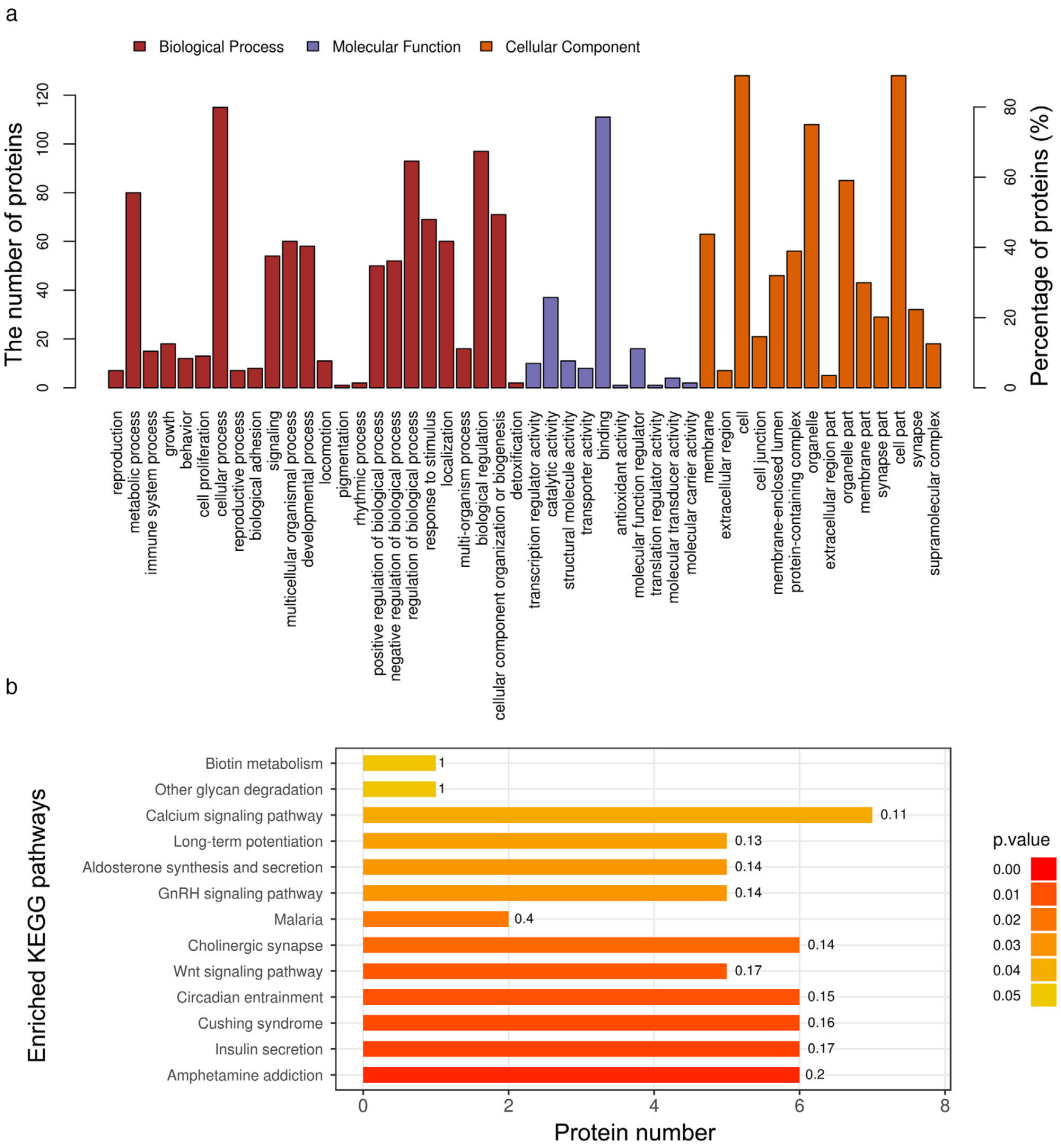
Gene ontology (GO) and Kyoto Encyclopedia of Genes and Genomes (KEGG) enrichment analyses of the differentially expressed phosphoproteins. (a) GO terms in three categories: biological processes (marked with red), molecular functions (marked with purple), and cellular component (marked with orange); y‐axis on the left: the number of annotated differentially expressed proteins; y‐axis on the right: the percentage of annotated proteins in all of the differentially expressed proteins. (b) The top 13 significantly enriched pathways annotated by KEGG analysis (false discovery rate < 0.05). The x‐axis indicates the number of proteins, with the corresponding KEGG pathway marked on the y‐axis. *p* < .05 represents statistically significant KEGG pathway enrichment

To further understand the functional distribution of the significant differentially expressed phosphoproteins identified from PS‐S offspring rats, the phosphoproteins were annotated and classified into CC, MF, and BP categories by Blast2GO (https://www.blast2go.com/) software (Figure 5(a)). CC analysis showed that protein phosphorylation events occurred in various types of the postsynaptic density, postsynaptic specialization, asymmetric synapses, neuron‐to‐neuron synapses, and internode regions of axons. The MF category reveals the overrepresentation of phosphoproteins involved in catalytic activity and binding. The latter includes structural constituents of the myelin sheath, basal transcription machinery binding, basal RNA polymerase II transcription machinery binding, RNA polymerase core enzyme binding, and calmodulin binding. The BP category provides information on the phosphoproteins overrepresented in response to progesterone, regulation of cell‐cell adhesion involved in gastrulation, positive regulation of chemokine secretion, chemokine (C‐C motif) ligand 2 secretion, and positive regulation of chemokine (C‐C motif) ligand 2 secretion. These proteins and relevant signaling pathways may play crucial roles in the depressive‐like behavior of offspring rats caused by PS.

The enrichment of KEGG pathways of different phosphorylation was analyzed by KAAS software. These phosphoproteins were found to be involved in many essential biological pathways, and the first five pathways included amphetamine addiction, insulin secretion, Cushing syndrome, and the circadian entrainment signaling pathway (Figure 5(b)). The first five pathways were related to nine proteins, including Adcy9, Apc, Cacna1c, Camk2a, Camk2b, Camk2g, Ctnnd2, Grin2a, and Stx1a. Additional research is needed to understand the precise role of these pathways in the depressive‐like behavior of offspring rats caused by PS.

## 4 DISCUSSION

Pregnancy is a special stage of life in women, and women are more susceptible to stress and often suffer from stress‐related disorders during this stage (Chen et al., 2020). Depression is the most common stress‐related disorder (Park et al., 2019). Prenatal restraint stress has been widely used to imitate daily repetitive stress events during pregnancy and to induce depression syndromes in animal models. In the present work, we implemented prenatal restraint stress on rats from the 14th to 20th day during gestation. SPT was used to screen offspring rats subjected to PS.

The SPT, OFT, and FST data showed consistent results that susceptible rat offspring showed remarkable depressive‐like behaviors, compared to CON rat offspring. These results are consistent with our previous work (Zhang et al., 2019) and have been confirmed by other laboratory findings (Kim et al., 2012; Tang et al., 2019). In the present work, quantitative phosphoproteomics revealed 144 different phosphoproteins between the PS‐S and CON groups. Among these different phosphoproteins, 100 were increased and 44 were decreased. GO enrichment analysis revealed that these different phosphoproteins in the first five enrichments of the CC, MF, and BP categories were involved in a total of 35 different phosphoproteins, and these phosphoproteins were mainly related to myelin, microtubule, and synapse‐associated proteins.

Myelin is a multilayered membrane sheath generated by the spiral wrapping of mature oligodendrocyte plasma membranes for rapid impulse propagation (Mount & Monje, 2017). Myelination is a dynamic process in the adult brain and is altered by experience (Mount & Monje, 2017). In recent years, myelin has been identified as a new player in the etiology and treatment of depression and stress‐related disorders (Boda, 2019). Consistent with this interpretation, the levels of N‐acetylaspartate (NAA), which maintains energetic integrity during myelination via oligodendroglial aspartoacylase (Francis et al., 2016), were reduced in the dorsolateral prefrontal white matter in major depressive disorder patients as detected by proton magnetic resonance spectroscopy (Wang et al., 2012). NAA is catabolized from NAA glutamate (NAAG) by glutamate carboxypeptidase II. Our previous work reported that PS could increase the level of NAAG in the hippocampus, which is the brain part most associated with depressive‐like behaviors in offspring rats caused by PS (Zhang et al., 2019). Prominent changes in tubulin expression levels are commonly found in disease‐specific regions such as the hippocampus and prefrontal cortex of psychiatric patients.

One study reported that depression was mediated by the AKT/GSK3β/CRMP‐2 pathway by changing the normal structure and function of the central nervous cell scaffold microtubule system (Wu et al., 2018). Stress hormone glucocorticoids (GCs) can activate GSK‐3, a kinase crucial to synapse weakening signals, which ultimately leads to phosphorylation of the microtubule‐associated protein tau, specifically at the serine 396 residue, and this is a causal factor in the GC‐mediated impairment of synaptic function (Yi et al., 2017). Tau protein is known to play an important role in maintaining microtubule assembly and stabilization. Another study reported that an aggregation of hyperphosphorylated tau protein appeared in the synapse of the hippocampus from C57BL/6 mice exposed to chronic unpredictable mild stress, and this effect could be reversed by ketamine, which is an N‐methyl‐D‐aspartate (NMDA) receptor antagonist that produces a rapid, long‐lasting, and potent antidepressant effect in patients suffering from major depression (Wen et al., 2019).

Our present results showed that PS could decrease the phosphorylation level of the microtubule‐associated protein tau and increase the phosphorylation level of the glutamate ionotropic receptor NMDA type subunit 2A. Collectively, both neurons and other CNS cells contribute to the pathogenesis of depressive disorders.

KEGG pathway analysis indicated that these different phosphoproteins were primarily involved in amphetamine addiction, insulin secretion, Cushing syndrome, circadian entrainment, and the Wnt signaling pathway. These main signaling pathways were related to the Adcy9, Apc, Cacna1c, Camk2a, Camk2b, Camk2g, Ctnnd2, Grin2a, and Stx1a proteins. According to previous reports, Cacna1c, Camk2a, Camk2g, Grin2a, and Stx1a are all candidate genes for depression (Calabrò et al., 2019; Y. Kim et al., 2016; Mitra et al., 2018; Wingo et al., 2018; Yawalkar et al., 2018). The correlation of Adcy9, Apc, Camk2b, Camk2g, and Ctnnd2 with the pathological mechanisms of depression was first reported in the present work. Extensive efforts are needed to determine the specific functions of these main proteins and the other proteins identified in the present work.

We preliminarily identified 144 different phosphoproteins between the PS‐S and CON groups, and these different phosphoproteins were mainly involved in myelin, microtubule, and synapse formation and plasticity in the hippocampus of susceptible offspring rats exposed to PS. The detailed mechanisms behind such perturbations should be studied.

## CONFLICT OF INTEREST

The authors declare no competing interests.

### PEER REVIEW

The peer review history for this article is available at https://publons.com/publon/10.1002/brb3.2233

## Supporting information



Supporting Information

## Data Availability

The data that support the findings of this study are available from the corresponding author upon reasonable request.
