# Supplementary material for: Phosphoproteomic profiling of the hippocampus of offspring rats exposed to prenatal stress
Source: Brain Behav. 2021 Sep 14;11(10):e2233. doi: 10.1002/brb3.2233 (PMC8553319; doi:10.1002/brb3.2233)
Supplement: Supplementary file 1 — Supporting Information [file BRB3-11-e2233-s001.docx]

| Supplementary Table 1. One-Sample Kolmogorov-Smirnov Test of data from SPT  **One-Sample Kolmogorov-Smirnov Test of data from SPT** | | | | | |
| --- | --- | --- | --- | --- | --- |
|  | | CON | PS-S | PS-M | PS-R |
| N | | 8 | 8 | 8 | 8 |
| Normal Parameters^a,b^ | Mean | .8065 | .4246 | .6431 | .7581 |
|  | Std. Deviation | .03996 | .04846 | .04938 | .04391 |
| Most Extreme Differences | Absolute | .168 | .208 | .269 | .218 |
|  | Positive | .168 | .208 | .165 | .218 |
|  | Negative | -.109 | -.159 | -.269 | -.173 |
| Test Statistic | | .168 | .208 | .269 | .218 |
| Asymp. Sig. (2-tailed) | | .200^c,d^ | .200^c,d^ | .093^c^ | .200^c,d^ |
| a. Test distribution is Normal. | | | | | |
| b. Calculated from data. | | | | | |
| c. Lilliefors Significance Correction. | | | | | |
| d. This is a lower bound of the true significance. | | | | | |

Supplementary Table 2. One-Sample Kolmogorov-Smirnov Test of data from OFT.

| **One-Sample Kolmogorov-Smirnov Test** **of data from OFT** | | | | | |
| --- | --- | --- | --- | --- | --- |
|  | | CON | PS-S | PS-M | PS-R |
| N | | 8 | 8 | 8 | 8 |
| Normal Parameters^a,b^ | Mean | 29161.0000 | 7386.3750 | 18190.6250 | 28490.6250 |
|  | Std. Deviation | 6242.68811 | 4170.43848 | 5809.46130 | 6789.74504 |
| Most Extreme Differences | Absolute | .205 | .200 | .180 | .243 |
|  | Positive | .205 | .200 | .096 | .217 |
|  | Negative | -.171 | -.180 | -.180 | -.243 |
| Test Statistic | | .205 | .200 | .180 | .243 |
| Asymp. Sig. (2-tailed) | | .200^c,d^ | .200^c,d^ | .200^c,d^ | .180^c^ |
| a. Test distribution is Normal. | | | | | |
| b. Calculated from data. | | | | | |
| c. Lilliefors Significance Correction. | | | | | |
| d. This is a lower bound of the true significance. | | | | | |

Supplementary Table 3. One-Sample Kolmogorov-Smirnov Test of data from FST.

| **One-Sample Kolmogorov-Smirnov Test of data from FST** | | | | | |
| --- | --- | --- | --- | --- | --- |
|  | | CON | PS-S | PS-M | PS-R |
| N | | 8 | 8 | 8 | 8 |
| Normal Parameters^a,b^ | Mean | 42.5000 | 96.8750 | 68.2500 | 40.8750 |
|  | Std. Deviation | 6.63325 | 18.59675 | 11.62202 | 7.16016 |
| Most Extreme Differences | Absolute | .147 | .288 | .174 | .133 |
|  | Positive | .147 | .288 | .174 | .133 |
|  | Negative | -.121 | -.188 | -.127 | -.090 |
| Test Statistic | | .147 | .288 | .174 | .133 |
| Asymp. Sig. (2-tailed) | | .200^c,d^ | .049^c^ | .200^c,d^ | .200^c,d^ |
| a. Test distribution is Normal. | | | | | |
| b. Calculated from data. | | | | | |
| c. Lilliefors Significance Correction. | | | | | |
| d. This is a lower bound of the true significance. | | | | | |

Supplementary Table 4. Phosphoserine and phosphothreonine motifs identified from 6790 differentially expressed phosphopeptides in PS-S and CON offspring rats (p < 0.05, PS vs. CON; one sample t-test).

**Supplementary Table 1 Motif analysis of phosphopeptides**

| **Number** | **Motif Logo** | **Motif** | **Score** | **Fold Increase** |
| --- | --- | --- | --- | --- |
| 1 | 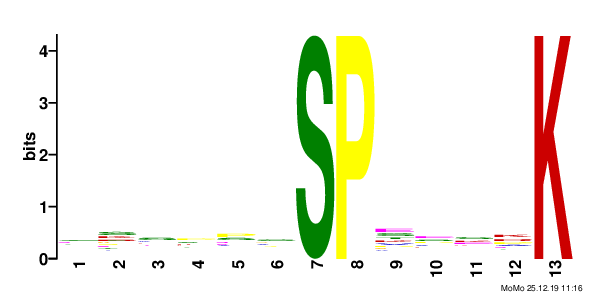 | ......_S_P....K | 32.00 | 8.1 |
| 2 | 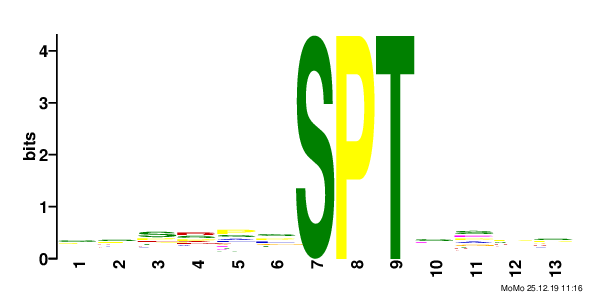 | ......_S_PT.... | 27.93 | 5.1 |
| 3 | 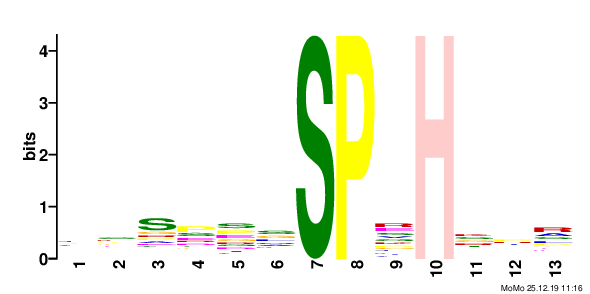 | ......_S_P.H... | 27.61 | 6.7 |
| 4 | 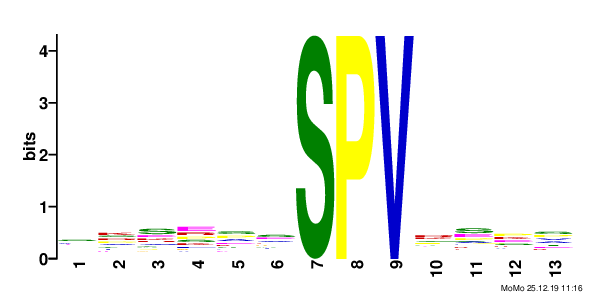 | ......_S_PV.... | 27.38 | 5.5 |
| 5 | 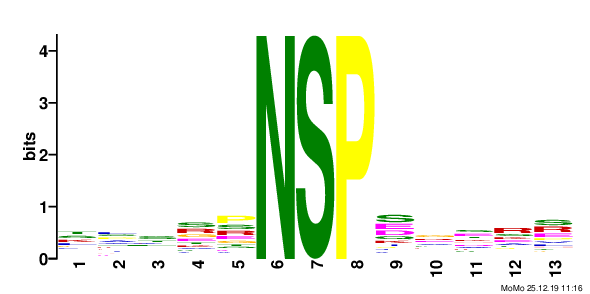 | .....N_S_P..... | 25.12 | 7.4 |
| 6 | 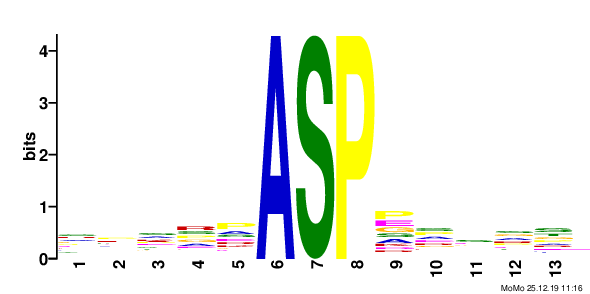 | .....A_S_P..... | 24.45 | 4.7 |
| 7 | 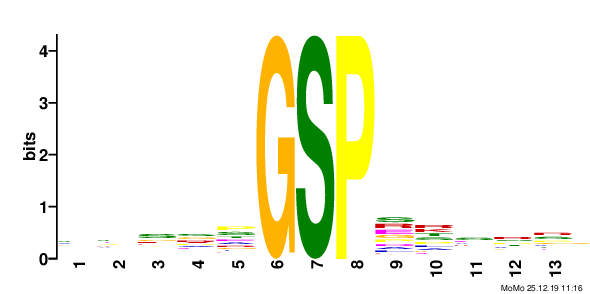 | .....G_S_P..... | 25.23 | 4.6 |
| 8 | 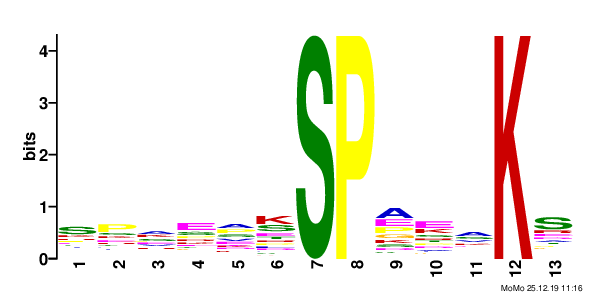 | ......_S_P...K. | 21.72 | 4.3 |
| 9 | 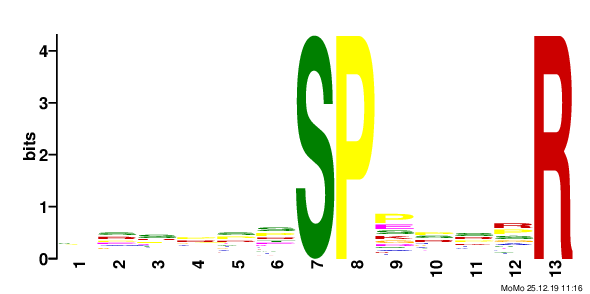 | ......_S_P....R | 21.41 | 4.1 |
| 10 | 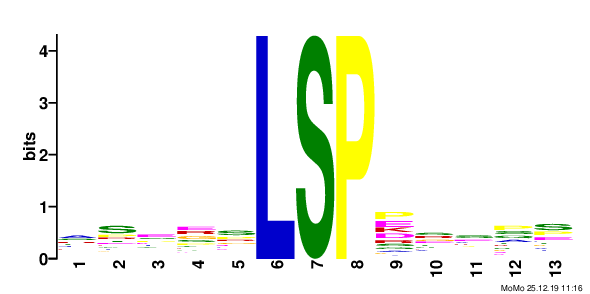 | .....L_S_P..... | 21.02 | 4.2 |
| 11 | 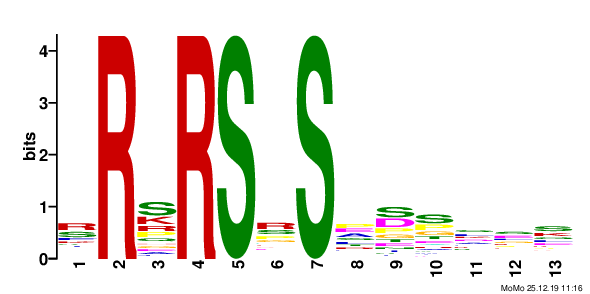 | .R.RS._S_...... | 45.02 | 10.9 |
| 12 | 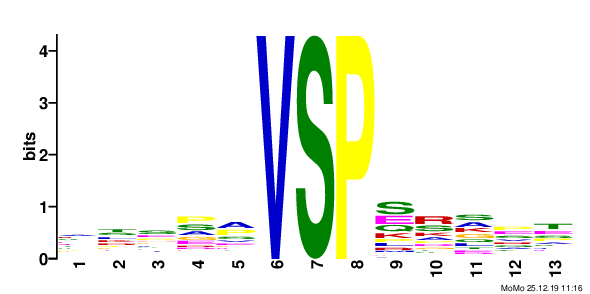 | .....V_S_P..... | 20.19 | 4.5 |
| 13 | 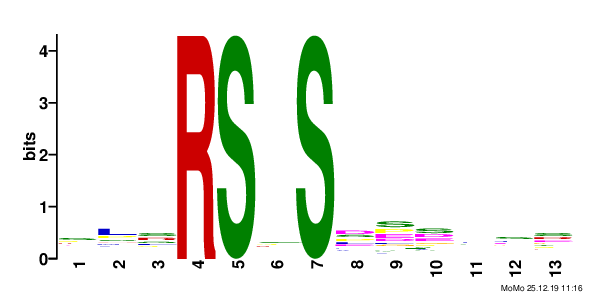 | ...RS._S_...... | 32.00 | 3.1 |
| 14 | 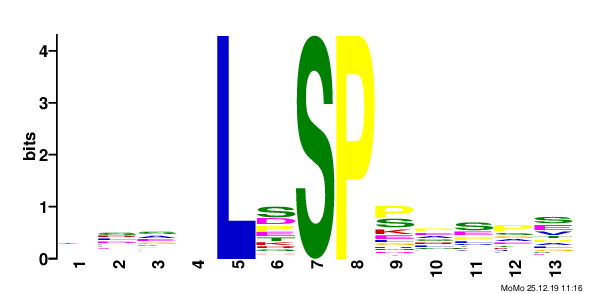 | ....L._S_P..... | 19.95 | 4.4 |
| 15 | 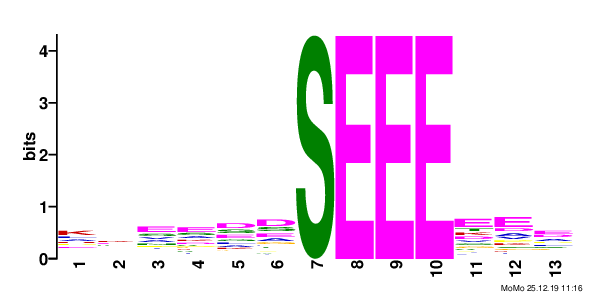 | ......_S_EEE... | 42.30 | 7.0 |
| 16 | 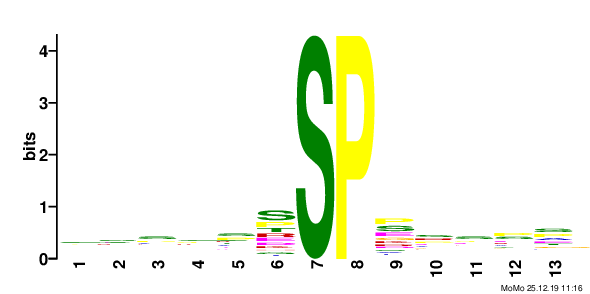 | ......_S_P..... | 16.00 | 2.7 |
| 17 | 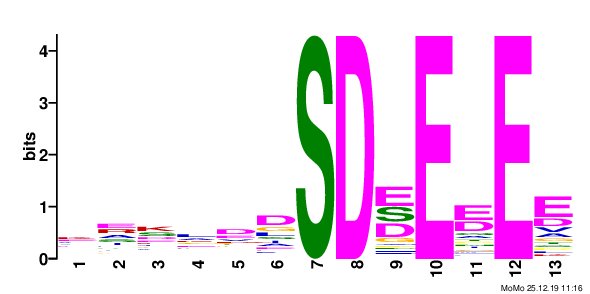 | ......_S_D.E.E. | 38.95 | 10.3 |
| 18 | 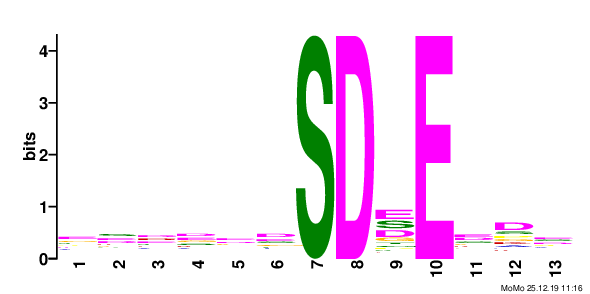 | ......_S_D.E... | 32.00 | 4.6 |
| 19 | 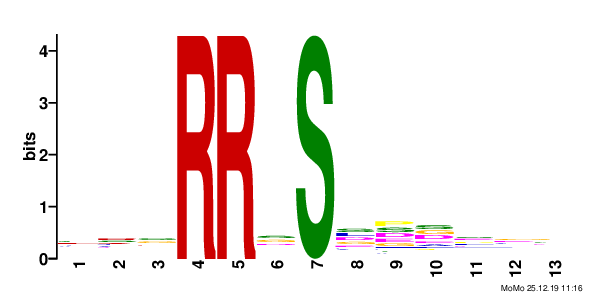 | ...RR._S_...... | 32.00 | 6.6 |
| 20 | 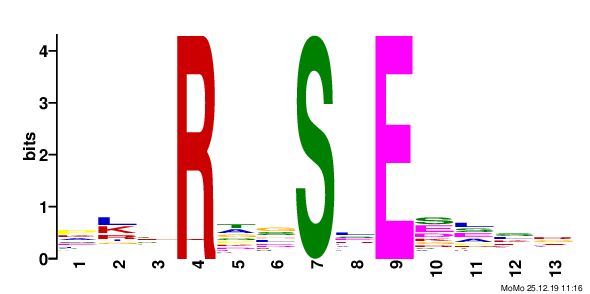 | ...R.._S_.E.... | 32.00 | 4.7 |
| 21 | 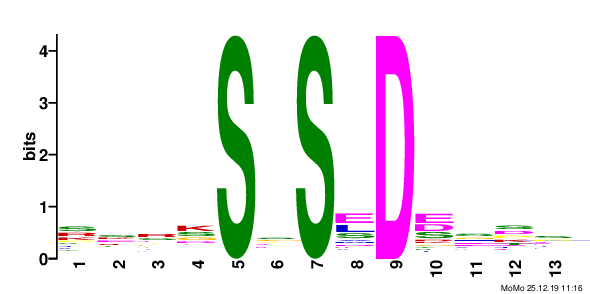 | ....S._S_.D.... | 25.15 | 2.7 |
| 22 | 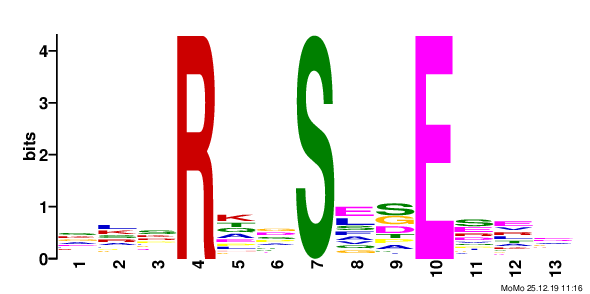 | ...R.._S_..E... | 29.86 | 4.5 |
| 23 | 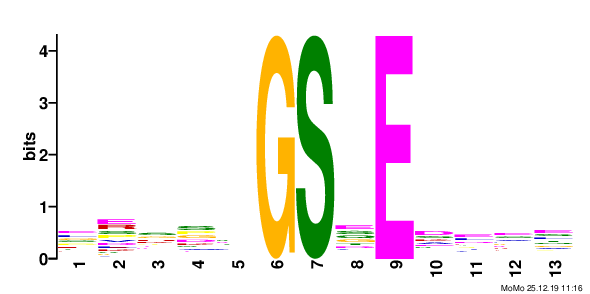 | .....G_S_.E.... | 20.01 | 2.5 |
| 24 | 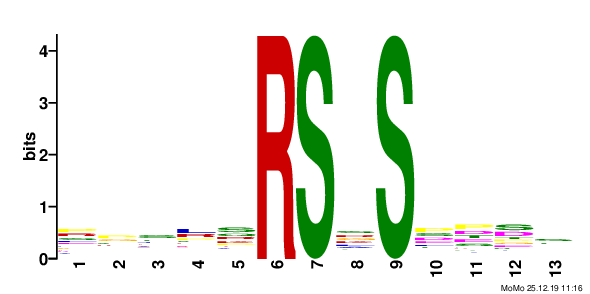 | .....R_S_.S.... | 25.20 | 2.6 |
| 25 | 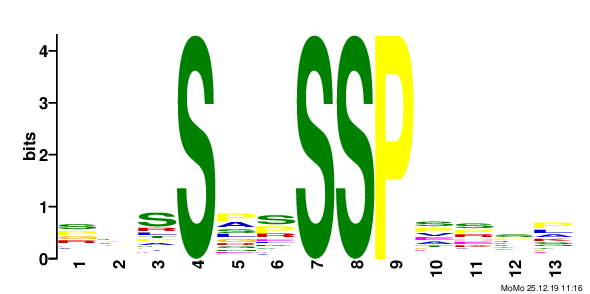 | ...S.._S_SP.... | 37.68 | 6.0 |
| 26 | 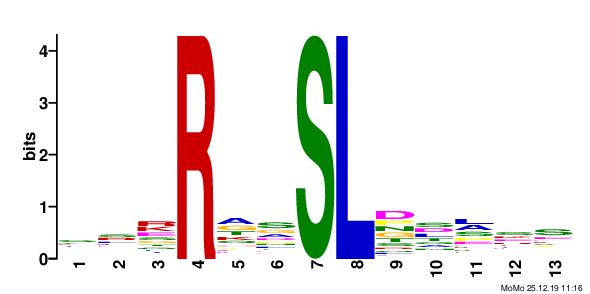 | ...R.._S_L..... | 25.23 | 3.9 |
| 27 | 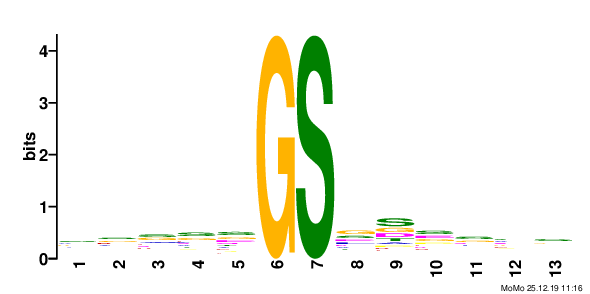 | .....G_S_...... | 16.00 | 1.6 |
| 28 | 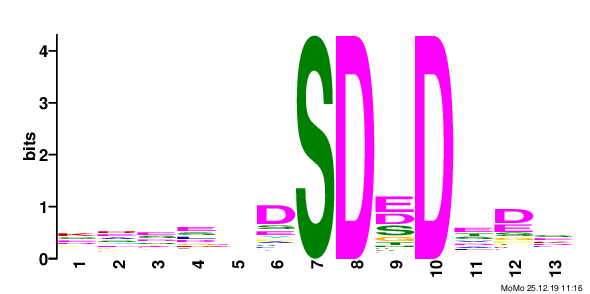 | ......_S_D.D... | 32.00 | 4.1 |
| 29 | 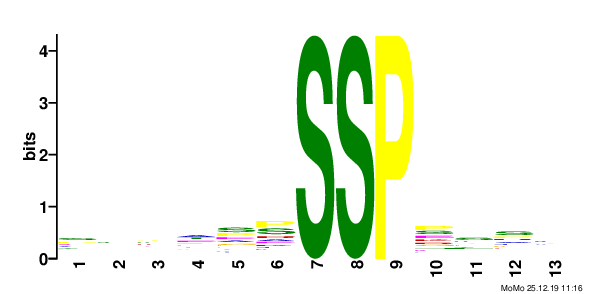 | ......_S_SP.... | 32.00 | 3.3 |
| 30 | 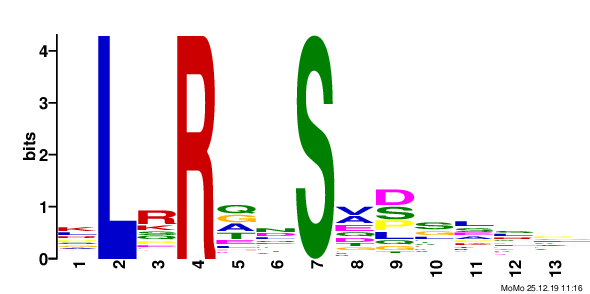 | .L.R.._S_...... | 29.63 | 5.9 |
| 31 | 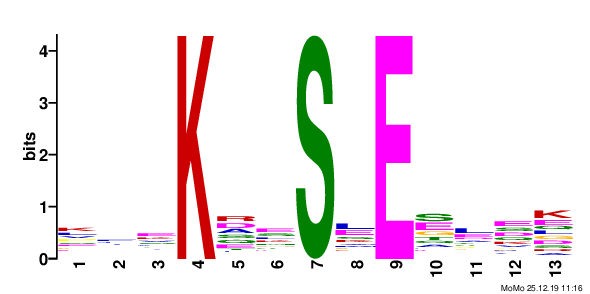 | ...K.._S_.E.... | 25.35 | 3.6 |
| 32 | 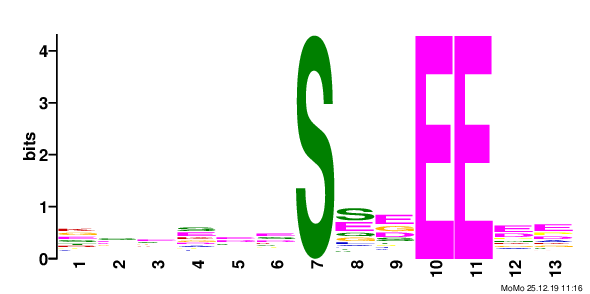 | ......_S_..EE.. | 20.32 | 2.7 |
| 33 | 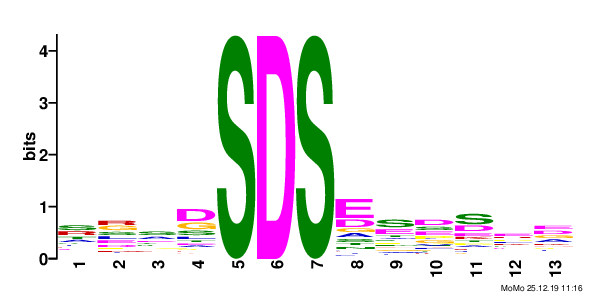 | ....SD_S_...... | 17.61 | 2.4 |
| 34 | 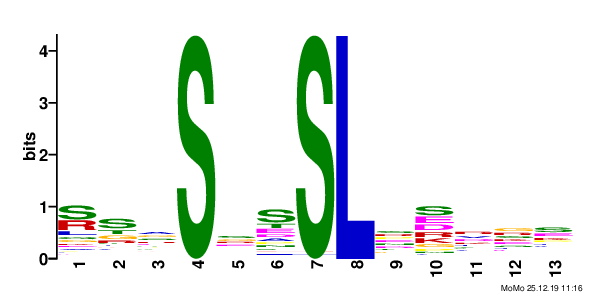 | ...S.._S_L..... | 17.05 | 2.7 |
| 35 | 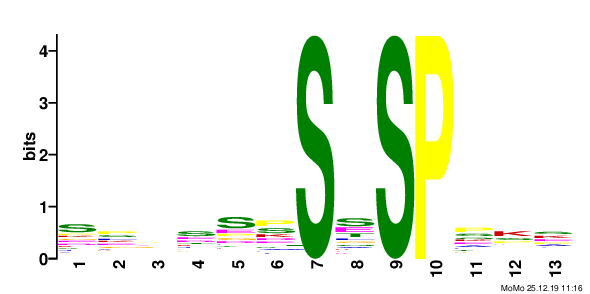 | ......_S_.SP... | 16.73 | 2.2 |
| 36 | 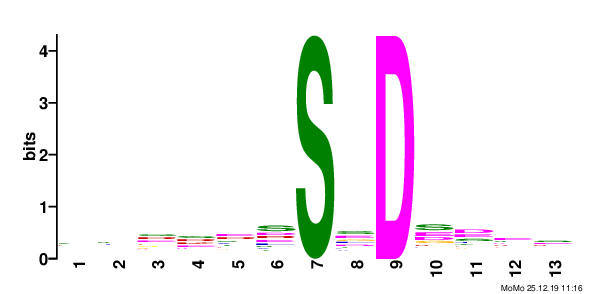 | ......_S_.D.... | 11.78 | 1.6 |
| 37 | 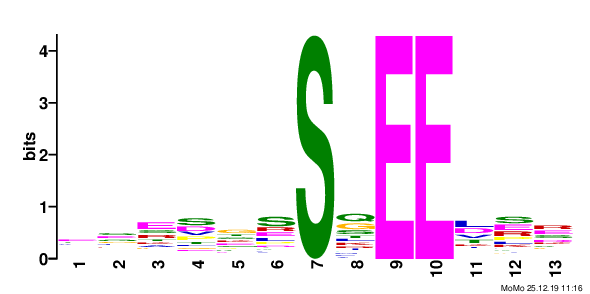 | ......_S_.EE... | 14.65 | 2.6 |
| 38 | 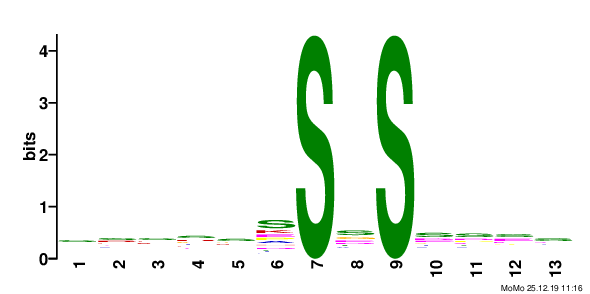 | ......_S_.S.... | 9.75 | 1.4 |
| 39 | 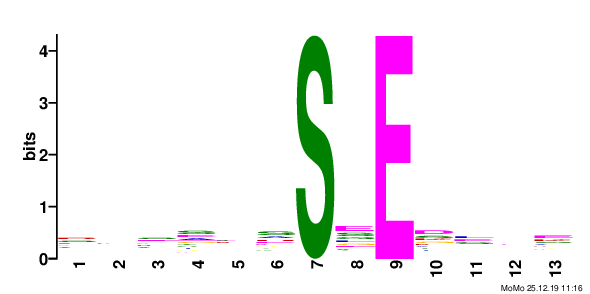 | ......_S_.E.... | 8.43 | 1.4 |
| 40 | 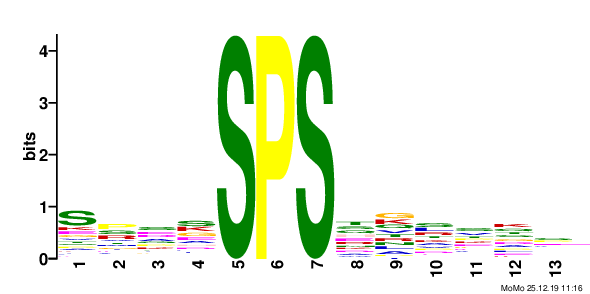 | ....SP_S_...... | 13.91 | 2.5 |
| 41 | 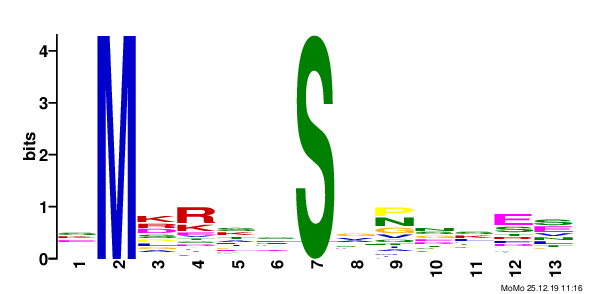 | .M...._S_...... | 7.71 | 2.3 |
| 42 | 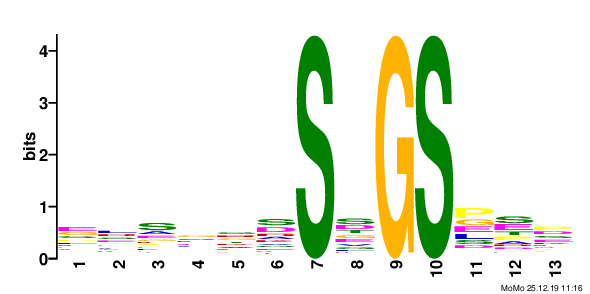 | ......_S_.GS... | 15.10 | 2.9 |
| 43 | 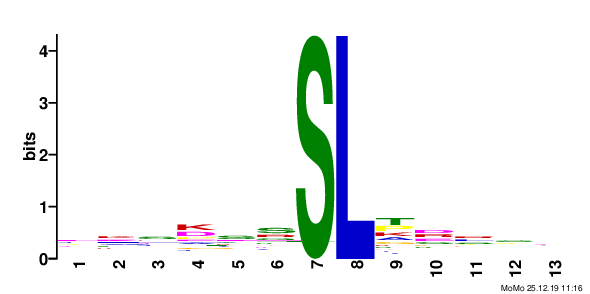 | ......_S_L..... | 7.63 | 1.7 |
| 44 | 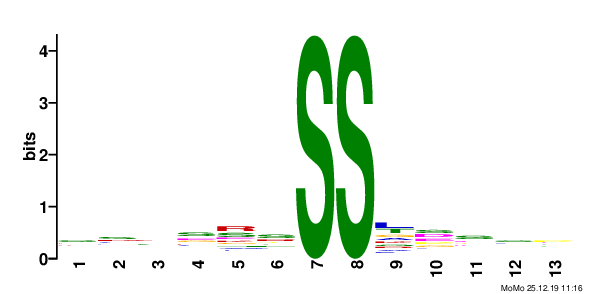 | ......_S_S..... | 7.25 | 1.4 |
| 45 | 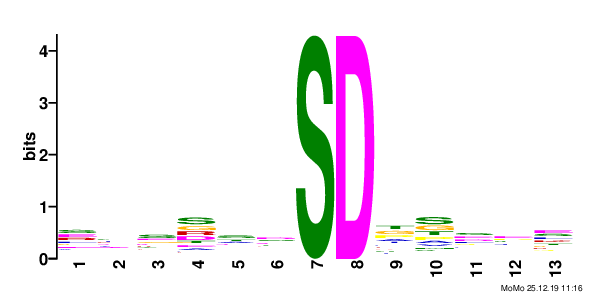 | ......_S_D..... | 8.50 | 1.7 |
| 46 | 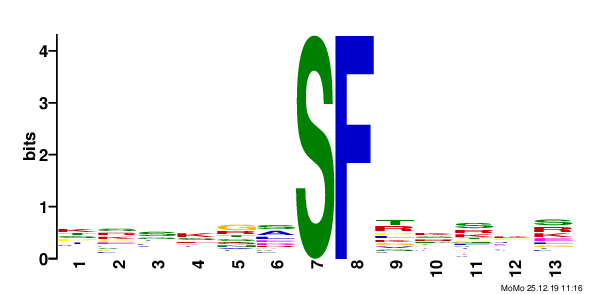 | ......_S_F..... | 6.28 | 2.1 |
| 47 | 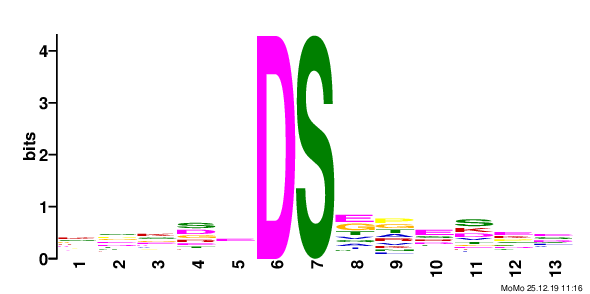 | .....D_S_...... | 5.21 | 1.6 |
| 48 | 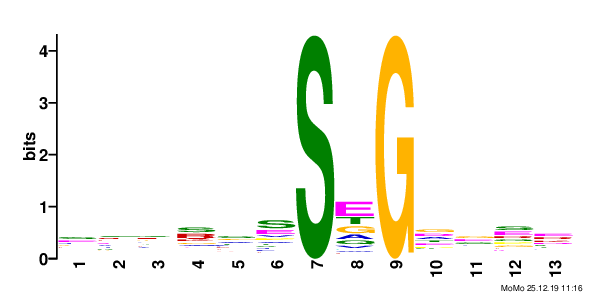 | ......_S_.G.... | 3.75 | 1.4 |
| 49 | 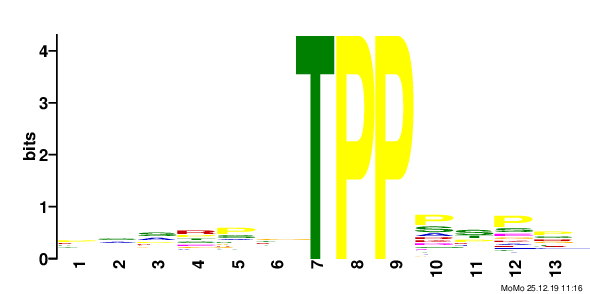 | ......_T_PP.... | 31.89 | 4.6 |
| 50 | 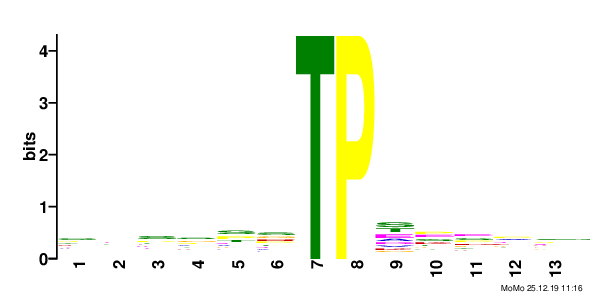 | ......_T_P..... | 16.00 | 1.9 |
| 51 | 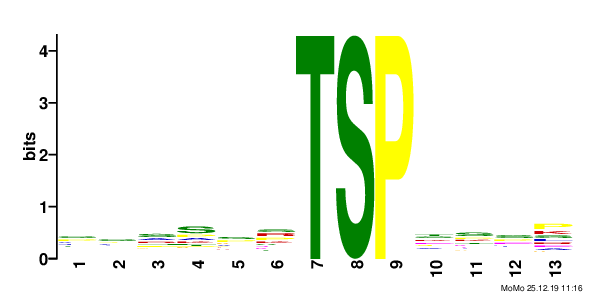 | ......_T_SP.... | 32.00 | 6.8 |
| 52 | 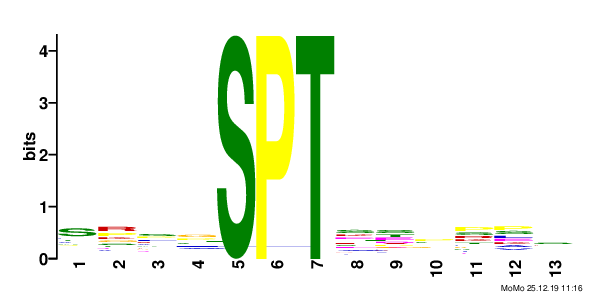 | ....SP_T_...... | 27.69 | 3.7 |
| 53 | 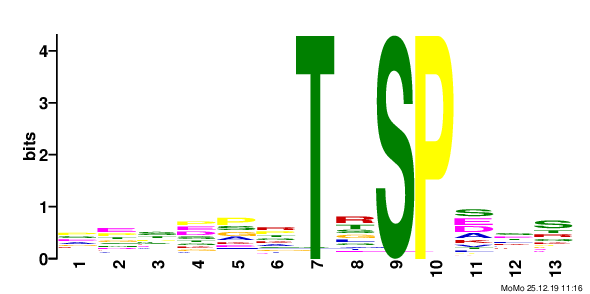 | ......_T_.SP... | 11.39 | 2.6 |
| 54 | 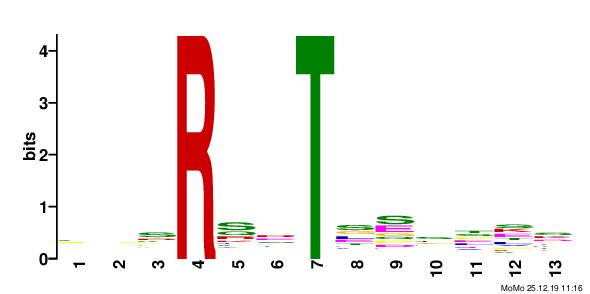 | ...R.._T_...... | 5.36 | 1.7 |
| 55 | 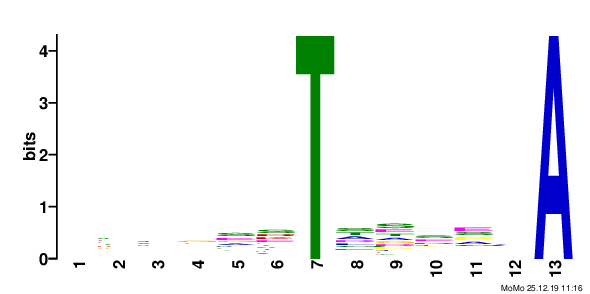 | ......_T_.....A | 4.07 | 1.6 |

Note: occurrences=50, significance=0.00018,

background=Ensembl_Rattus_29107_20190628
